# Diversity, Distribution and Host Blood Meal Analysis of Adult Black Flies (Diptera: Simuliidae) from Thailand

**DOI:** 10.3390/insects15010074

**Published:** 2024-01-21

**Authors:** Bhuvadol Gomontean, Waraporn Jumpato, Komgrit Wongpakam, Ubon Tangkawanit, Wannachai Wannasingha, Isara Thanee, Zubaidah Ya’cob, Pairot Pramual

**Affiliations:** 1 Department of Biology, Faculty of Science, Mahasarakham University, Maha Sarakham 44150, Thailand; bhuvadol.g@msu.ac.th (B.G.); waraporn.a2536@gmail.com (W.J.); wannachai.wan@msu.ac.th (W.W.); isara.th@msu.ac.th (I.T.); 2 Walai Rukhavej Botanical Research Institute, Mahasarakham University, Maha Sarakham 44150, Thailand; komwongpa@gmail.com; 3 Department of Entomology and Plant Pathology, Faculty of Agriculture, Khon Kaen University, Khon Kaen 40002, Thailand; ubonta@kku.ac.th; 4 Higher Institution Centre of Excellence, Tropical Infectious Diseases Research and Education Centre (TIDREC), Universiti Malaya, Kuala Lumpur 50603, Malaysia; zyacob@um.edu.my

**Keywords:** insect vector, *Simulium*, hematophagous insect, DNA barcode

## Abstract

**Simple Summary:**

Black flies are small-sized (<6 mm) blood-sucking insects belonging to the family Simuliidae of the order Diptera. More than 2400 species have been recorded globally, and approximately 6% (145 species) are found in Thailand. Many black fly species are pests to humans and other animals. They also transmit pathogens such as viruses, protozoa and filarial nematodes to humans and other animals, including economically significant livestock. Therefore, knowledge of species the diversity and distribution of wild adults, as well as host blood sources, provides important information required to prevent problems related to black fly biting. In this study, we collected 7706 wild adult black flies over a seven-year period (2017–2023) from diverse natural habitats in Thailand. In total, 16 black fly taxa were recorded; one of them, *Simulium yvonneae* Takaoka and Low, is a new record for the country. The majority of the specimens were *Simulium asakoae* Takaoka and Davies complex, contributing 74% of the total number of specimens collected. This species predominantly occurs in forest and urban habitats. The second most abundant species, *S. chumpornense* Takaoka and Kuvangkadilok (16% of the total specimens collected) was predominant in domestic animal shelters. Host blood meal identification using molecular approaches revealed four vertebrate host species: humans, chickens, turkeys and water buffalo.

**Abstract:**

Understanding the factors associated with the species diversity and distribution of insect vectors is critically important for disease epidemiology. Black flies (Diptera: Simuliidae) are significant hematophagous insects, as many species are pests and vectors that transmit pathogens to humans and other animals. Ecological factors associated with black fly species distribution have been extensively examined for the immature stages but are far less well explored for the adult stage. In this study, we collected a total of 7706 adult black fly specimens from various locations in forests, villages and animal shelters in Thailand. The integration of morphology and DNA barcoding revealed 16 black fly taxa, including *Simulium yvonneae*, a species first found in Vietnam, which is a new record for Thailand. The most abundant species was the *Simulium asakoae* complex (*n* = 5739, 74%), followed by *S. chumpornense* Takaoka and Kuvangkadilok (n = 1232, 16%). The *Simulium asakoae* complex was dominant in forest (3786 of 4456; 85%) and village (1774 of 2077; 85%) habitats, while *S. chumpornense* predominated (857 of 1175; 73%) in animal shelter areas. The *Simulium asakoae* complex and *S. nigrogilvum* Summers, which are significant pests and vectors in Thailand, occurred at a wide range of elevations, although the latter species was found mainly in high (>1000 m) mountain areas. *Simulium chumpornense*, *S. nodosum* Puri and the *S. siamense* Takaoka and Suzuki complex occurred predominately in low (<800 m)-elevation areas. *Simulium furvum* Takaoka and Srisuka; *S. phurueaense* Tangkawanit, Wongpakam and Pramual; and *S.* nr*. phurueaense* were only found in high (>1000 m) mountain areas. A host blood meal analysis revealed that the *S. asakoae*; *S. chamlongi* Takaoka and Suzuki; *S. nigrogilvum*; *S. chumpornense*; and the *S. striatum* species group were biting humans. This is the first report of the latter two species biting humans. We also found that *S. chumpornense* was biting turkeys, and *S. chamlongi* was biting chickens, which are new host blood sources recorded for these species. In addition, we found that the *S. feuerborni* Edwards complex was biting water buffalo, which is the first report on the biting habits of this species.

## 1. Introduction

Black flies (Diptera: Simuliidae) are small hematophagous insects that can be found in every continent except Antarctica [1]. Up to 2022, there have been 2415 species recorded globally: 2398 living and 17 fossils [2,3]. Approximately 10–20% of these black fly species are pests, and many of these also transmit pathogens to humans and other animals [1]. The most significant disease for which black fly species are vectors is human onchocerciasis or river blindness, which is caused by the filarial nematode *Onchocerca volvulus*; at least 26 species of the black fly genus *Simulium* are vectors [1]. There are 14.6 million infected people who already have skin disease, and 1.15 million have experienced vision loss [4]. Black flies also transmit other filarial nematodes and blood protozoa of the genera *Leucocytozoon* and *Trypanosoma* to wild and domestic animals [1].

In total, 145 black fly species assigned to six subgenera (*Asiosimulium* Takaoka and Choochote; *Daviesellum* Takaoka and Adler; *Gomphostilbia* Enderlein; *Montisimulium* Rubstsov; *Nevermannia* Enderlein; and *Simulium* Latreille) have been recorded in Thailand [5]. Two species, *S. nigrogilvum* and *S. nodosum*, are considered to be human pests in the northern region [1,6]. *Simulium nigrogilvum* is also a vector of the *Onchocerca* sp. [7,8,9,10,11]. In addition, *S. chumpornense* and the *S. asakoae* complex are possible vectors of blood protozoa of the genera *Leucocytozoon*, the causative agent of leucocytozoonosis in poultry [1], and avian *Trypanosoma* [12,13,14], which does not typically cause disease.

Understanding the factors associated with the species diversity, distribution and abundance of vector species is necessary for disease epidemiology [15], as this information can be used to predict the risk areas where vector species are most likely to occur [16]. However, most of the ecological studies of black flies have been based on the immature stages (e.g., [17,18,19]). Factors associated with the diversity, distribution and abundance of the adult black flies are much less well explored. Investigations in Thailand have revealed that season and elevation are major factors associated with species diversity and abundance [9,20,21]. However, these studies were restricted to a particular location [8,9,21,22] or focused on a few species [20]. Because diversity and abundance can be affected by geographic location and habitat topography [23], wider geographic sampling is needed.

Another aspect of black fly biology that remains underexplored is biting behavior. Knowledge of host blood sources is important for determining the pest and vector potential of particular species [24]. The females of approximately 90% of black fly species need a blood meal for egg maturation [1]. However, biting behavior is only known in 10 of 145 (7%) black fly species in Thailand [25]. Seven species (*S. asakoae* complex, *S. nigrogilvum*, *S. nodosum*, *S. chamlongi*, *S. doipuiense* complex, *S. tenebrosum* complex and *S. umphangense*) are human biters; two species (*S. nodosum* and *S*. *nakhonense*) are water buffalo biters; and two species (*S. asakoae* complex and *S. chumpornense*) are chicken biters [8,14,22,26,27].

In this study, we examined adult black fly biodiversity and distribution in northern and northeastern Thailand across > 700 km transects in three different habitat categories: natural forests, villages and animal shelters. We also used molecular methods to identify host blood meals from blood-fed females. Information presented in this study will be useful for understanding the factors associated with the species distribution of wild adult black flies. Knowledge of host blood sources is essential for determining potential pest and vector species.

## 2. Materials and Methods

### 2.1. Specimen Collection and Species Identification

**Figure 1:**
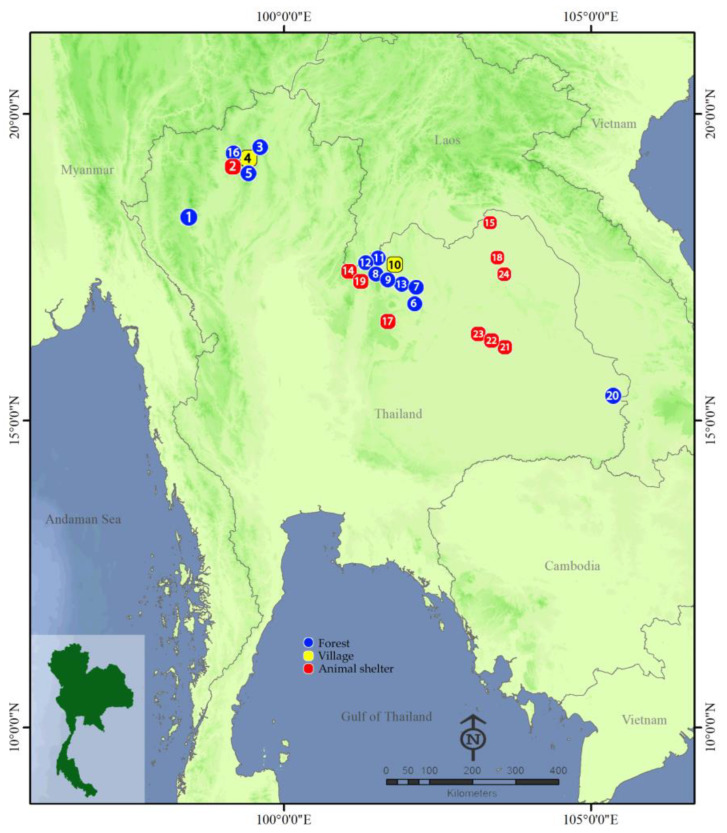
Map showing 24 sampling locations of wild adult black flies from Thailand used in this study. Details of sampling locations are included in Table 1.

A total of 35 collections were made from 24 sampling sites in north and northeastern Thailand between October 2017 and May 2023 (Table 1 and Figure 1). Specimens were collected from three habitat categories: natural forests (F), villages (V) and animal shelters (AS, a place where farmers keep their livestock). Wild adults were collected using a figure-eight motion net sweeping swept randomly in the air at 0.5–2.0 m above the ground. The sweep nets used had three-part telescopic handles with a total extended length of 120 cm and a 39 cm hoop diameter. Collections were made early in the morning (6:00–8:00 am) and late afternoon (16:00–18:00 pm), which are the times when adult black flies actively search for host blood meals [9,22]. Specimens were pooled in plastic vials containing 80% ethanol and stored at −20 °C in a freezer until use. Species identification of black fly specimens was performed using the most recent morphological keys to black flies in Thailand [28]. Because many black fly species are morphologically similar, representative specimens of morphologically identified species were also subjected to molecular identification using DNA barcoding based on mitochondrial cytochrome c oxidase I (COI) sequences.

### 2.2. Molecular Analysis

#### 2.2.1. DNA Barcode

Genomic DNA was extracted from the whole individual specimen using the GF-1 Nucleic Acid DNA extraction kit (Vivantis Technologies Sdn. Bhd., Malaysia). A 650 bp fragment of the COI gene was amplified using primers LCO1490 (5′-GGTCAACAAA TCATAAAGATATTGG-3′) and HCO2198 (5′-TAAACTTCAGGGTGACAAAAAATC A-3′). The PCR reaction conditions described in Tangkawanit et al. [29] were used to amplify the COI gene. PCR products were checked with 1% agarose gel electrophoresis and were purified using the PureDireX PCR CleanUp & Gel Extraction kit (Bio-Helix, Taiwan, China). Purified PCR products were sequenced at ATCG Company Limited (Thailand Science Park, Pathumthani, Thailand) using the same primers as for PCR.

#### 2.2.2. Host Blood Meal Identification

Female specimens were first examined under a compound microscope to detect a blood meal. Only blood-engorged female specimens were used for molecular identification of host blood sources. In total, 54 specimens were used for blood meal source identifications. The same DNA extraction method was used for blood meal source identification. Mitochondrial cytochrome *b* (cyt *b*) of the vertebrate host blood was amplified using the primers L14841 and H15149 [30] and PCR reaction conditions as described in Malmqvist et al. [31]. PCR purification and sequencing were as described in the DNA barcoding study except for the primers used for sequencing cyt *b*.

#### 2.2.3. Data Analysis

To support morphological taxonomy, the COI sequences (accession nos. PP043244-PP043310) of adult black flies were used for species identification using the identification engine in the Barcode of Life Data Systems (BOLD, https://www.boldsystems.org/index.php/IDS_OpenIdEngine) (accessed on 25 November 2023). Species identification in BOLD is based on the threshold value of sequence similarity between query and reference sequences available in the database [32]. If the query shows <1% sequence divergence from the reference sequence, species identification results. In cases where more than one species has reference sequences showing a <1% match with the query, the identification is considered ambiguous. For species that have no matching sequences (>3% divergence from the closest reference sequence) in BOLD, the genetic similarities of the specimens obtained in the present study were compared with reports in GenBank, and phylogenetic analysis based on the neighbor-joining (NJ) and maximum likelihood (ML) methods was conducted. The NJ tree was calculated in MEGA X [33] using the Kimura 2-parameter (K2P) model with branch support estimated using bootstrapping with 1000 replicates. The ML tree was also inferred in MEGA X using the general time-reversible (GTR) model with gamma distribution + invariant sites (G + I). Brach support was estimated using bootstrapping with 1000 replicates. Identification of the host blood sources was performed by determining the sequence similarity of vertebrate cyt *b* gene sequences using the Basic Local Alignment Search Tool (BLAST) (https://blast.ncbi.nlm.nih.gov/Blast.cgi, accessed on 25 November 2023) in the National Center for Biotechnology Information database.

## 3. Results

### 3.1. Species Diversity and Abundance

A total of 7706 adult specimens were collected. Four subgenera (*Asiosimulium*, *Gomphostilbia*, *Nevermannia* and *Simulium*) were identified based on morphological diagnostic characters, including katepisternum-bearing hairs (*Gomphostilbia*); cibariums with distinct groups of medially spinous processes (*Asiosimulium*); and claws with large basal teeth (*Nevermannia*), with only a small or without basal tooth (*Simulium*) [28]. Morphological identification at the species level revealed 14 nominal species. The most diverse subgenus was *Simulium*, represented by seven species. Three species were recorded for subgenus *Gomphostilbia* and two species for *Asiosimulium* and *Nevermannia* (Table 2).

A total of 67 representative COI specimens for these morphospecies were used for DNA-based species identification. DNA barcoding revealed that 36 specimens from eight species agreed with morphological identifications. The remaining sequences from seven morphospecies were ambiguous, as the COI barcoding sequences were closely related to two or more species (Table 2). Specimens morphologically identified as the *S. doipuiense* complex, but with COI gene sequences ambiguously identified, were treated as the *S. doipuiense* complex/*S. tenebrosum* complex. The ambiguity of the COI-determined identity of these species was noted in a previous study [25]. However, the colorations of the hind tibiae and barsitarsi of the female can be used to clearly differentiate the *S. doipuiense* complex from the *S. tenebrosum* complex [34]. Other species with COI sequences ambiguously identified in BOLD were the *S. asakoae* complex, *S. chumpornense*, the *S. feuerborni* complex, *S. nodosum*, *S. fenestratum* and the *S. striatum* species group.

**Figure 2:**
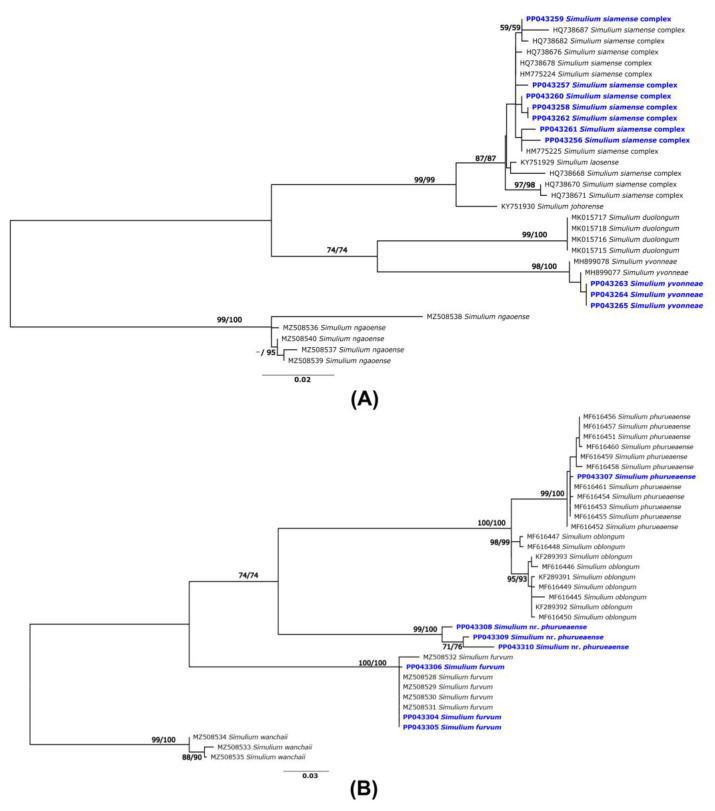
ML tree based on mitochondrial COI gene sequences of the adult specimens collected in this study (blue) and those reported in GenBank: (**A**) the *S. batoense* species group of the subgenus *Gomphostilbia* and (**B**) subgenus *Asiosimulium*. Bootstrap numbers based on 1000 replications for ML and NJ analyses are shown near the branches.

Three specimens were morphologically identified as the *S. siamense* complex, but the DNA barcoding analysis indicated that they were *S. yvonneae*, which was originally described in Vietnam [35]. Phylogenetic analyses of other species of the *S. batoense* species group supported an interpretation that three specimens from northeastern Thailand were *S. yvonneae*, as they formed a clade with this species with strong (>99%) bootstrap support (Figure 2A). Seven COI sequences, three from *S. furvum* and four from *S. phurueaense*, had no match references in BOLD because the COI sequences of these species have not yet been included in BOLD. However, using BLAST in the NCBI’s GenBank revealed that all specimens of *S. furvum* had >98% sequence similarity compared with the COI sequences of this species reported previously [25]. Only one of four COI sequences of specimens morphologically identified as *S. phurueaense* showed high (>99%) sequence similarity with this species as recorded in GenBank [29]. Three sequences had only 90% maximum similarity with a member of the subgenus *Asiosimulium* (*S*. *oblongum*, *S. phurueaense*, *S. furvum*), although they were collected from locations close (<2 km) to the type locality of *S. phurueaense*. Phylogenetic analysis also supports the conclusion that these specimens are different species, as they formed a separate and well-supported clade (Figure 2B). Therefore, we refer to this species as *S*. nr. *phurueaense* in this study.

Based on morphology and molecular identifications, adults of 16 black fly species were collected in the present study. Among these species, *S. yvonneae* originally described in Vietnam [35], was identified based on the COI barcoding sequences and is a new record in Thailand. The number of species taken at each sampling site varied between one and eight (Table 1). The most diverse (eight species) and most abundant (3657 specimens) sampling site was at Ban Pang Bong, Chiang Mai Province, in northern Thailand. Two other sampling sites, Song Khon Waterfall and Ban Pa Chan Tom, both from Phu Ruea District, Loei Province, in northeastern Thailand, also had relatively high species diversity, each with six species (Table 1). The most abundant species was the *S. asakoae* complex (n = 5739, 74.47%), followed by *S. chumpornense* (n = 1232, 16.01%) and *S. nigrogilvum* (n = 327, 4.23%). The remaining species were present at very low relative abundance (<1%) except for *S. nodosum*, for which a total of 120 (1.56%) specimens were collected (Table 2).

### 3.2. Patterns of Species Distributions in Different Habitat Types and Elevations

The majority (n = 4456, 57.83%) of the specimens were collected from natural forests, followed by those from villages (n = 2077, 26.95%) and animal shelters (n = 1175, 15.25%) (Table 3). Only two species (the *S. asakoae* complex and *S. chumpornense*) were found in all three habitat types. Eight species (*S. furvum*, *S. phurueaense*, *S.* nr. *phurueaense*, the *S. feuerborni* complex, *S. chamlongi*, *S. fenestratum*, the *S. doipuiense* complex and *S. yuphae*) were found exclusively in the forest areas. Two other species were also found mainly in the forest areas, *S. nigrogilvum* (319 of 326) and the *S. striatum* species group (41 of 51), although the remaining specimens were collected from other habitat types. Six species (the *S. asakoae* complex, *S. chumpornense*, the *S. siamense* complex, *S. yvonneae*, *S. nigrogilvum* and *S. nodosum*) were found in villages. Among these, only two species, the *S. asakoae* complex and *S. chumpornense*, were found in high numbers (1774 for *S. asakoae* complex and 857 for *S. chumpornense*). Six species (the *S. asakoae* complex, *S. chumpornense*, the *S. siamense* complex, *S. aureohirtum*, *S. nodosum* and the *S. striatum* species group) were found in or around the animal shelters. Species with a majority of specimens collected from animal shelters were *S. chumpornense* (68%), the *S. siamense* complex (56%) and *S. nodosum* (97%).

A relatively high (48.99%) number of adult black fly specimens were collected from habitats at elevations > 1000 m above sea level (asl) (Table 4). Elevations from 400 to 600 m asl and 600 to 800 m asl also supported large numbers of individuals (17.92% and 27.09%, respectively). One species (S. *aureohirtum*) was found across all elevations except 200–400 m and 800–1000 m asl. *Simulium chumpornense* and the *S. asakoae* complex were also found across a wide range of elevations, ranging from <200 m asl to 800 m asl and from 400 m asl to 1200 m asl, respectively. Four species, *S. furvum*, *S. phurueaense*, *S. chamlongi* and the *S. doipuiense* complex, were found only at >1000 m asl. *Simulium nodosum* was found only at elevations between 400 and 800 m asl.

### 3.3. Host Blood Meal Identifications

**Figure 3:**
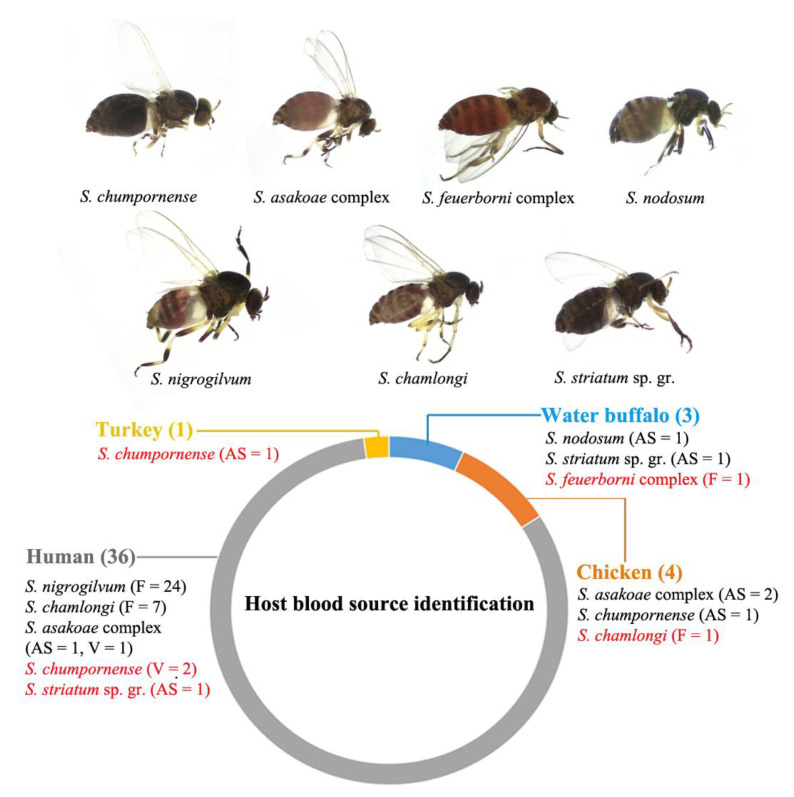
Photographs of blood-fed females of black fly species collected in the present study and the result of host blood meal identification based on BLAST results of the mitochondrial cyt *b* gene sequence. Habitat types (F, forest; AS, animal shelter; V, village) where blood-fed females collected and the number of specimens are indicated following the species name. Red indicates a new host blood source record.

A total of 54 blood-fed female specimens from seven species (the *S. asakoae* complex (n = 6), *S. chumpornense* (n = 5), the *S. feuerborni* complex (n = 2), *S. chamlongi* (n = 8), the *S. striatum* sp. gr. (n = 2), *S. nodosum* (n = 2) and *S. nigrogilvum* (n = 29)) were collected. These specimens were used for the molecular identification of host blood sources using mitochondrial cyt *b* sequences. In total, host blood sources were successfully identified for 44 of 54 (81%) blood-fed specimens (Figure 3). The majority of the host blood sources were human (36 of 44, 81%). The human-biter species were the *S. asakoae* complex, *S. chamlongi*, *S. nigrogilvum*, *S. chumpornense* and the *S. striatum* sp. gr. Among these, *S. chumpornense* and the *S. striatum* sp. gr. are reported here for the first time as feeding on human blood. Most human-biter specimens (31 of 36) were collected from a forest habitat that was located close (approximately 1 km) to a village. Two specimens, one from *S. asakoae* and another from the *S. striatum* sp. gr., were collected from animal shelters that were also located close (approximately 3 km) to a village.

Six specimens, three from *S. asakoae* complex, two from *S. chumpornense* and one from *S. chamlongi*, were found to be chicken biters. The finding that *S. chamlongi* fed on chicken blood is a new host blood record for this species. Three specimens, each from the *S. feuerborni* complex, *S. nodosum* and the *S. striatum* sp. gr., were biters of water buffalo. This host blood meal identification for the *S. feuerborni* complex is the first report of feeding behavior for this species in Thailand. One specimen of *S. chumpornense* was found to be feeding on turkeys (*Meleagris gallopavo*), and this is a new host blood record for this species.

## 4. Discussion

### 4.1. Diversity, Abundance and Cryptic Diversity in Wild Adult Black Flies

In this study, we found that adult specimens collected from Ban Pang Bong, Chiang Mai Province, in northern Thailand, were the most diverse (8 of 16) and most abundant (3657 of 7706). This location, situated in a mountainous area at high elevation (>1000 m asl), generally experiences cold temperatures throughout the year. There are also many streams of diverse size, velocity and streambed particles (personal observation), and some of these flow year-round (according to local people). These conditions can support the high species diversity of black flies [17,18,36]. This sampling location is also close to a village (approximately 1 km) where several blood sources, including humans and their livestock, are available for adult females. The availability of diverse immature habitats and host blood sources thus supports the high diversity and abundance of black flies in this location.

Specimens of the *Simulium asakoae* complex were the most abundant species found in this study. The majority (4866 of 5739) were collected from two sampling sites in the northern region, Ban Huai Mo and Ban Pang Bong (both in Chiang Mai Province). The high abundance of adult *S. asakoae* complex in the northern region agrees with a previous study by Ishii et al. [9], who collected >6000 adult specimens of this species from a location also situated in Chiang Mai Province. Ecological studies of both immature and adult stages revealed that this species occurs in wide elevation ranges [18,21,36]. The breeding sites of the *S. asakoae* complex are small, slow-flowing streams with small streambed particles (e.g.*,* mud, sand, gravel) [36]. These habitat characteristics are common in mountainous areas in the northern region of Thailand [18,36]; consequently, the *S. asakoae* complex is common and abundantly found in this region. 

Black flies are very small-sized insects, and many closely related species are morphologically similar or undistinguishable in certain life stages. Therefore, an integrated approach is preferable for correct species identification [3,37]. In this study, three female specimens collected from Loei Province in northeastern Thailand were morphologically identified as the *S. siamense* complex but were molecularly determined to be *S. yvonneae*, a species of the same species group (the *S. batoense* species group), which has been recorded only in Vietnam [2,35]. The adult stages of the *S. siamense* complex and *S. yvonneae* are very similar, and they are only distinguishable at the pupal and larval stages [35]. Despite their morphological similarity, the COI barcoding sequences clearly differentiated these species with >9.30% sequence divergence [35]. Sequences of the specimens suspected as *S. yvonneae* collected from northeastern Thailand in the present study were >99% similar to the COI sequences of this species reported by Takaoka et al. [35], which were obtained from specimens collected from the type locality. Phylogenetic analysis also supported that these three specimens from Loei Province were *S. yvonneae*. Therefore, this species possibly occurs in Thailand and, thus, is probably a new record for the country. The further collection of the immature stages in which it is possible to distinguish *S. yvonneae* from the *S. siamense* complex will be helpful in confirming the existence of the former species in Thailand. A similar situation was reported previously for *S. myanmarense*, a species that was originally described from Myanmar but is suspected to occur in northern Thailand based on the DNA barcode sequence similarity of specimens morphologically identified as *S. asakoae* [38]. Later morphological and molecular analyses have confirmed the existence of *S. myanmarense* in Thailand [39]. Based on these results, we recommended that using both morphology and DNA barcoding is necessary for fully understanding black fly biodiversity.

In addition to the finding of a possible new record of a black fly species in Thailand, we also detected cryptic genetic diversity in specimens identified as *S. phurueaense* despite specimens being collected from a site very close (<2 km) to the type locality of this species. There are two possible explanations for this finding of cryptic genetic diversity. First, it is possible that those specimens showing high genetic divergence from *S. phurueaense* might reflect the presence of *Wolbachia* DNA or the nuclear mitochondrial pseudogene (*numt*), as both situations can produce genetically divergent lineages [40,41]. Further investigation using other genetic markers, such as those from nuclear genes, will be useful in testing this hypothesis. Alternatively, the cryptic genetic divergence detected in *S. phurueaense* might represent another unidentified species of the subgenus *Asiosimulium* that is morphologically similar to *S. phurueaense*. Note that we only examined female specimens, which potentially show very similar morphological characteristics between closely related species, such as between *S. phurueaense* and *S. oblongum* [29]. Therefore, a further investigation of other life stages of *S*. nr. *Phurueaense* is required to test this hypothesis.

### 4.2. Distribution along the Elevation Gradients and Habitat Types of Wild Adult Black Flies

Previous studies have shown that different black fly species reach their abundance peak in different seasons [9,21,22]. This is possibly related to the availability of suitable breeding sites in different seasons [20]. In this study, we collected most specimens (89.92%) during the cold season. This is due, in part, to the sampling bias of our collection dates, where most (20 of 35) collections were conducted during the cold season.

Srisuka et al. [21] categorized black fly species collected along an elevation gradient from 400 to 2500 m at Doi Inthanon mountain, in northern Thailand, into seven categories. Most (9 of 16) of the species collected in the present study agree with the elevation categories of Srisuka et al. [21]. The *Simulium asakoae* complex and *S. nigrogilvum* belong to the wide-range elevation category, and *S. chumpornense* and the *S. siamense* complex belong to the low-elevation groups. The *S. doipuiense* complex and the *S. feuerborni* complex were placed in the mid-elevation zone. However, while *S. aureohirtum* and *S. yuphae* were placed in the mid-elevation zone (1400–2200 m asl) by Srisuka et al. [21], we also collected these species at much lower elevations. The lowest elevation habitat for *S. aureohirtum* collected in the present study was 120 m asl, and those for *S. yuphae* were at 730 m asl. Therefore, these species occur across a wide range of elevations. The occurrence of the wild adults of these species across a wide elevation range agrees with previous reports on immature habitats where *S. aureohirtum* can be found in locations as low as 24 m asl [42], as well as 680 m asl for *S. yuphae* [25]. The *Simulium striatum* sp. gr., which was assigned to the low-elevation group (<700 m asl) by Srisuka et al. [21], was also collected from a high-elevation site (1080 m asl) in the present study. Therefore, members of the *S. striatum* sp. gr. Are distributed over a wide elevation range.

Most (4456 of 7706, or 57.83%) adult specimens were collected from natural forest habitats. All species except the *S. siamense* complex were found in forest areas. Eight species (*S. furvum*, *S. phurueaense*, *S.* nr*. Phurueaense*, the *S. feuerborni* complex, *S. chamlongi*, *S. fenestratum*, the *S. doipuiense* complex and *S. yuphae*) were found exclusively in the forest. The majority of the specimens of *S. aureohirtum*, *S. nigrogilvum* and the *S. striatum* sp. gr. were also collected from forest areas. In the urban habitats, only six species were found. Among these, only the two common species (the *S. asakoae* complex and *S. chumpornense*) were abundant (>100 individuals). These two species were also found in high numbers in and around the animal shelter areas. Another species for which most of the collected specimens were found around animal shelters was *S. nodosum*, as 117 of 120 were collected from this habitat. The finding that the *S. asakoae* complex, *S. chumpornense* and *S. nodosum* occurred in high numbers in the animal shelter areas is consistent with previous knowledge stating that these species bite domestic animals such as chickens [14] and water buffalo [8]. Finding the *S. striatum* sp. gr. in animal shelter areas also agrees with a previous report on the biting habits of another member of this species group, *S. nakhonense*, which feeds on water buffalo blood [8].

### 4.3. Host Blood Meal Identification

More than half (29 of 54) of the blood-fed specimens collected in this study were from *S. nigrogilvum*, a human pest species [1,6]. This is because most of the specimens of this species were collected from a forest close to a village (Ban Pang Bong, Doi Saket District, Chiang Mai Province) where local people experience problems with this human-biting species. All host blood sources for *S. nigrogilvum* were identified as human. Another species with a considerable number (eight) of blood-fed females was *S. chamlongi*, which was also collected from the same location as *S. nigrogilvum*. This species was already known to be a human biter [22], and the results of the molecular identification of host blood meals support the previous finding, as seven of eight specimens were human blood. However, one specimen was identified as chicken blood, and this is a new host blood record for *S. chamlongi*. The host blood meal identifications for *S. nodosum* revealed that this species feeds on water buffalo blood, thus supporting previous findings [8]. Identifications of the host blood sources of the *S. asakoae* complex as human and chicken and those of *S. chumpornense* as chicken also support previous reports of the biting habits of these species [14]. However, we also found that *S. chumpornense* fed on humans based on the molecular identification of the host blood source. The host blood sources of the *S. striatum* sp. gr. specimens were molecularly identified as water buffalo and humans. The finding showing that members of the *S. striatum* sp. gr. were feeding on water buffalo agrees with a previous report stating that *S. nakhonense*, a member of the *S. striatum* sp. gr., is a buffalo biter [8]. However, another blood-fed specimen of the *S. striatum* sp. gr., collected around cattle pens from Loei Province, had fed on a human. This is another new host blood source record for this species. We also report, for the first time, the biting habits of the *S. feuerborni* complex, which feeds on water buffalo.

Black fly species with claws that possess a large basal tooth are usually ornithophilic; that is, they feed on avian species because the bifid claw seems to be an adaptation for grasping bird feathers [43]. However, based on our host blood meal identifications, the species that have bifid claws also feed on mammals. Morphologically, the *S. feuerborni* complex belongs to the subgenus *Nevermannia*, in which the female has claws with a large basal tooth [44]. *Simulium chumpornense* also feeds on humans according to the results of this study, although the majority of its host blood sources were from avian species (chickens and turkeys). Finding that species with bifid claws (i.e., claws with a large basal tooth) also feed on mammals is not unexpected. The *Simulium asakoae* complex is within the subgenus *Gomphostilbia*, which also possesses claws with a large basal tooth and feeds on humans [22] in addition to avian species such as chickens. *Simulium chamlongi* has long been known as a human biter [22]. In this study, we found that this species also bites chickens. Regarding the morphological characteristic of the claws, *S. chamlongi* has a subbasal tooth [44], but whether this subbasal tooth is involved in ornithophilic behavior remains to be explored.

## 5. Conclusions

In conclusion, we report a new record of *Simulium* species in Thailand, *S. yvonneae*, which, so far, has only been found in Vietnam. We also report, for the first time, the biting habits of members of the subgenus *Nevermannia* in Thailand in the *S. feuerborni* complex, which feeds on water buffalo. In addition, new host blood sources for *S. chumpornense* (human and turkey), *S. chamlongi* (chicken) and the *S. striatum* sp. gr. (human) are recorded. This information is crucial for determining vectorial capacity and disease epidemiology involving these hematophagous insects [24]. Because there are still many black fly species in Thailand that have not been examined for biting behavior, further effort should be focused on this topic. We have learned from the present study that a considerable number of blood-fed specimens can be found in or near animal shelters and villages. Therefore, further study can be focused on these particular areas, which will be helpful in determining the biting behavior of black flies and possibly related to the disease epidemiology of livestock.

## Figures and Tables

**Table 1:** Sampling location, number by sex (male, female) and female blood-fed specimens of wild adult black flies collected in Thailand between October 2017 and May 2023.

Location (Type ^a^)	Coordinate	Elevation (m)	Date	Species	Male	Female	Blood-Fed	Total
1. Bo Kaeo Pine Tree Garden, Hot, Chiang Mai (F)	18.156452 N, 98.388855 E	1051	20 October 2017	*S. furvum*	-	52	-	52
				*S. asakoae* complex	-	40	-	40
2. Ban Pang Fan, Doi Saket, Chiang Mai (F)	19.016450 N, 99.302972 E	592	24 January 2020	*S. asakoae* complex	-	4	-	4
3. Mae Chedi Check Point, Wiang Pa Pao, Chiang Rai (F)	19.085784 N, 99.408942 E	774	24 January 2020	*S. asakoae* complex	-	5	-	5
4. Ban Huai Mo, Doi Saket, Chiang Mai (V)	19.040857 N, 99.335092 E	735	24 January 2020	*S. asakoae* complex	-	1634	1	1635
				*S. nigrogilvum*	-	7	-	7
				*S. nodosum*	-	3	-	3
5. Ban Pang Bong, Doi Saket, Chiang Mai (F)	18.985475 N, 99.335629 E	1080	25 January 2020	*S. asakoae* complex	6	2375	-	2381
				*S. nigrogilvum*	-	60	-	60
				*S. chamlongi*	-	17	-	17
			10 February 2023	*S. asakoae* complex	1	849	-	850
				*S. striatum* species group	1	28	-	29
				*S. nigrogilvum*	-	230	29	259
				*S. fenestratum*	-	2	-	2
				*S. chamlongi*	-	43	8	51
				*S. doipuiense* complex	-	3	-	3
				*S. feuerborni* complex	-	2	2	4
				*S. aureohirtum*	1	0	-	1
6. Suan Hom Waterfall, Nong Hin, Loei (F)	17.047652 N, 101.761805 E	558	8 August 2020	*S. aureohirtum*	8	10	-	18
7. Ban Kok Bok, Wang Saphung, Loei (F)	17.392824 N, 101.571823 E	278	8 August 2020	*S. chumpornense*	-	1	-	1
8. Ban Song Khon, Phu Ruea, Loei (F)	17.360647 N, 101.396537 E	653	8 August 2020	*S. chumpornense*	-	2	-	2
				*S. asakoae* complex	-	9	-	9
				*S. aureohirtum*	6	27	-	33
			5 March 2022	*S. asakoae* complex	-	12	-	12
9. Song Khon waterfall, Phu Ruea, Loei (F)	17.353900 N, 101.404916 E	730	8 August 2020	*S. asakoae* complex	-	9	-	9
			5 March 2022	*S. striatum* species group	-	3	-	3
				*S. asakoae* complex	1	195	-	196
				*S. fenestratum*	-	2	-	2
			21 January 2023	*S. asakoae* complex	-	25	1	26
			4 February 2023	*S. chumpornense*	-	43	-	43
				*S. asakoae* complex	-	80	-	80
				*S. yvonneae*	-	1	-	1
				*S. yuphae*	-	2	-	2
10. Ban Nong Bua, Phu Ruea, Loei (V)	17.448202 N, 101.343799 E	597	9 August 2020	*S. chumpornense*	-	84	-	84
				*S. siamense* complex	-	6	-	6
				*S. asakoae* complex	-	18	-	18
			21 March 2021	*S. yvonneae*	-	1	-	1
				*S. asakoae* complex	-	9	-	9
				*S. chumpornense*	1	26	1	28
			6 March 2022	*S. asakoae* complex	-	91	-	91
				*S*. *chumpornense*	-	82	-	82
				*S. siamense* complex	-	2	-	2
			5 February 2023	*S. asakoae* complex	-	21	-	21
				*S. chumpornense*	-	89	-	89
				*S. yvonneae*	-	1	-	1
11. Phu Ruea, Loei (F)	17.502904 N, 101.349973 E	1217	9 August 2020	*S.* nr*. phurueaense*	-	18	-	18
12. Hin Sam Chan waterfall, Phu Ruea, Loei (F)	17.499911 N, 101.336031 E	1156	9 August 2020	*S. asakoae* complex	-	1	-	1
				*S. phurueaense*	-	5	-	5
13. Phu Ruea Highland Agricultural Experiment Station, Loei (F)	17.297928 N, 101.410619 E	927	20 March 2021	*S. striatum* species group	-	3	-	3
				*S. asakoae* complex	-	13	-	13
				*S. doipuiense* complex	-	2	-	2
				*S.* nr*. phurueaense*	-	1	-	1
			21 January 2023	*S. asakoae* complex	-	159	1	160
				*S. striatum* species group	-	6	-	6
14. Ban Pa Chan Tom, Phu Ruea, Loei (AS)	17.457579 N, 101.335994 E	590	22 January 2023	*S. asakoae* complex	-	102	2	104
				*S. striatum* species group	-	9	1	10
				*S. chumpornense*	-	2	-	2
			4 February 2023	*S. asakoae* complex	-	36	-	36
				*S. chumpornense*	-	484	-	484
				*S. siamense* complex	-	9	-	9
				*S. aureohirtum*	-	2	-	2
			5 February 2023	*S. asakoae* complex	-	18	-	18
				*S. chumpornense*	-	117	1	118
				*S. nodosum*	-	1	-	1
				*S. siamense* complex	-	1	-	1
15. Ban Non Du, Rattanawapi, Nong Khai (AS)	18.193845 N, 103.321423 E	150	2 January 2023	*S. chumpornense*	2	200	-	202
16. Ban Pang Dang, Doi Saket, Chiang Mai (AS)	18.997487 N, 99.280149 E	484	11 February 2023	*S. asakoae* complex	-	10	-	10
				*S. nodosum*	-	114	2	116
				*S. nigrogilvum*	-	1	-	1
				*S. striatum* species group	-	-	1	1
17. Phu Khiao Wildlife Husbandry Research Station, Khon San, Chaiyaphum (AS)	16.492121 N, 101.715468 E	629	5 May 2023	*S. chumpornense*	-	9	-	9
				*S. asakoae* complex	-	1	-	1
18. Ban Thon, Chareon Sin, Sakon Nakhon (AS)	17.688918 N, 103.532279 E	170	18 February 2023	*S. chumpornense*	-	10	1	11
19. Ban Pla Ba, Phu Ruea, Loei (AS)	17.381556 N, 101.350172 E	630	20 March 2021	*S. asakoae* complex	-	9	1	10
				*S. chumpornense*	-	15	1	16
20. Khong Chiam, Ubon Ratchathani (F)	15.316529 N, 105.512555 E	120	12 Nov 2022	*S. chumpornense*	-	47	1	48
				*S. aureohirtum*	-	2	-	2
21. Ban Kerng, Maha Sarakham (AS)	16.219851 N, 103.330584 E	150	14 February 2021	*S. chumpornense*	-	2	-	2
22. Ban Don Suan, Kantharawichai, Maha Sarakham (AS)	16.257045 N, 103.266384 E	150	15 February 2021	*S. chumpornense*	-	3	-	3
23. Ban Wai, Kantharawichai, Maha Sarakham (AS)	16.307443 N, 103.188846 E	150	22 February 2021	*S. chumpornense*	-	2	-	2
24. Waritchaphum, Sakon Nakhon (AS)	17.242257 N, 103.574797 E	190	10 November 2022	*S. chumpornense*	-	6	-	6
Total					27	7625	54	7706

^a^ F, forest; V, village; AS, animal shelter.

**Table 2:** List of morphospecies; results of species identification using DNA barcoding sequences; number by sex (male, female); and female blood-fed specimens of each black fly taxon in Thailand collected between October 2017 and May 2023.

Subgenus/Morphospecies	BOLD Identification (n)	Male	Female	Blood-Fed	Total
***Asiosimulium* Takaoka and Choochote**					
*S. furvum* Takaoka and Srisuka	*S. furvuma* (3) ^a^	-	52	-	52 (0.67)
*S. phurueaense* Tangkawanit,	*S. phurueaensea* (1) ^a^,	-	5	-	5 (0.06)
Wongpakam and Pramual	*S*. nr. *phurueaense* (3)		19	-	19 (0.25)
***Gomphostilbia* Enderlein**					
*S. asakoae* Takaoka and Davies complex	*S. asakoae* (6), *S. asakoae/* *S. myanmarense* (1)	8	5725	6	5739 (74.47)
*S. chumpornense* Takaoka and Kuvangkadilok	*S. chumpornense* (2), *S. kuvangkadilokae*/ *S. chumpornense/S. piroonae* (3)	3	1224	5	1232 (15.99)
*S. siamense* Takaoka and Suzuki complex	*S. siamense* complex (7)	-	18	-	18 (0.23)
	*S. yvonneae* (3)	-	3	-	3 (0.04)
***Nevermannia* Enderlein**					
*S. aureohirtum* Brunetti	*S. aureohirtum* (4)	15	41	-	56 (0.73)
*S. feuerborni* Edwards complex	*S. feuerborni* complex (1), *S. feuerborni* complex*/* *S. fruticosum* (1)	-	2	2	4 (0.05)
***Simulium* Latreille s. str.**					
*S. chamlongi* Takaoka and Suzuki	*S. chamlongi* (10)	-	60	8	68 (0.88)
*S. doipuiense* Takaoka and Choochote complex	*S. doipuiense* complex*/* *S. tenebrosum* complex*/* *S. rufibasis* (5)	-	5	-	5 (0.06)
*S. nigrogilvum* Summers	*S. nigrogilvum* (8)	-	298	29	327 (4.24)
*S. nodosum* Puri	*S. nodosum/S. shirakii* (3)	-	118	2	120 (1.56)
*S. striatum* species group	*S. chiangmaiense/S. nakhonense/* *S. quinquestriatum* (2)	1	49	2	52 (0.66)
*S. yuphae* Takaoka and Choochote	*S. yuphae* (2)	-	2	-	2 (0.03)
*S. fenestratum* Edwards	*S. fenestratum/S. chaliowae* (2)	-	4	-	4 (0.05)
**Total**		**27**	**7625**	**54**	**7706**

^a^ No match sequence in BOLD; species identification was based on sequence similarity using BLAST in GenBank.

**Table 3:** Occurrence of 16 adult black fly species in Thailand in different habitat types collected between October 2017 and May 2023.

Species	Habitat Type			Total
	Forest	Village	Animal Shelter	
*Asiosimulium*				
*S. furvum*	52	-	-	52
*S. phurueaense*	5	-	-	5
*S.* nr*. phurueaense*	19	-	-	19
*Gomphostilbia*				
*S. asakoae* complex	3786	1774	179	5739
*S. chumpornense*	94	283	855	1232
*S. siamense* complex	0	8	10	18
*S. yvonneae*	1	2	-	3
*Nevermannia*				
*S. aureohirtum*	54	-	2	56
*S. feuerborni* complex	4	-	-	4
*Simulium*				
*S. nigrogilvum*	320	7	-	327
*S. nodosum*	-	3	117	120
*S. chamlongi*	68	-	-	68
*S. striatum* species group	41	-	11	52
*S. fenestratum*	4	-	-	4
*S. doipuiense* complex	5	-	-	5
*S. yuphae*	2	-	-	2
Total (16 taxa)	4455 (57.81)	2077 (26.95)	1174 (15.23)	7706

**Table 4:** Occurrence of 16 adult black fly species in Thailand at different elevations collected between October 2017 and May 2023.

Species	Elevation (m)					
	<200	200–400	400–600	600–800	800–1000	>1000
*Asiosimulium*						
*S. furvum*	-	-	-	-	-	52
*S. phurueaense*	-	-	-	-	-	5
*S.* nr*. phurueaense*	-	-	-	-	1	18
*Gomphostilbia*						
*S. asakoae* complex	-	-	311	1983	173	3272
*S. chumpornense*	274	1	903	54	-	-
*S. siamense* complex	-	-	18	-	-	-
*S. yvonneae*	-	-	2	1	-	-
*Nevermannia*						-
*S. aureohirtum*	2	0	20	33	-	1
*S. feuerborni* complex	-	-	-	-	-	4
*Simulium*						
*S. nigrogilvum*	-	-	-	7	-	320
*S. nodosum*	-	-	117	3	-	-
*S. chamlongi*	-	-	-	-	-	68
*S. striatum* species group	-	-	11	3	9	29
*S. fenestratum*	-	-	-	2	2	-
*S. doipuiense* complex	-	-	-	-	-	5
*S. yuphae*	-	-	-	2	-	-
Total (%)	276 (3.58)	1 (0.01)	1382 (17.93)	2088 (27.10)	185 (2.40)	3774 (48.97)

## Data Availability

The data generated during the study are reported in the manuscript.
